# Clinical value of miR-198-5p in lung squamous cell carcinoma assessed using microarray and RT-qPCR

**DOI:** 10.1186/s12957-018-1320-y

**Published:** 2018-02-02

**Authors:** Yue-ya Liang, Jia-cheng Huang, Rui-xue Tang, Wen-jie Chen, Peng Chen, Wei-luan Cen, Ke Shi, Li Gao, Xiang Gao, An-gui Liu, Xiao-tong Peng, Gang Chen, Su-ning Huang, Ye-ying Fang, Yong-yao Gu

**Affiliations:** 1 Department of Pathology, First Affiliated Hospital of Guangxi Medical University, Nanning, Guangxi People’s Republic of China; 2 Department of Radiotherapy, First Affiliated Hospital of Guangxi Medical University, Nanning, Guangxi People’s Republic of China

**Keywords:** MiR-198-5p, Expression, Lung squamous cell carcinoma, Target genes, Bioinformatics

## Abstract

**Background:**

To examine the clinical value of miR-198-5p in lung squamous cell carcinoma (LUSC).

**Methods:**

Gene Expression Omnibus (GEO) microarray datasets were used to explore the miR-198-5p expression and its diagnostic value in LUSC. Real-time reverse transcription quantitative polymerase chain reaction was used to evaluate the expression of miR-198-5p in 23 formalin-fixed, paraffin-embedded (FFPE) LUSC tissues and corresponding non-cancerous tissues. The correlation between miR-198-5p expression and clinic pathological features was assessed. Meanwhile, putative target messenger RNAs of miR-198-5p were identified based on the analysis of differentially expressed genes in the Cancer Genome Atlas (TCGA) and 12 miRNA prediction tools. Subsequently, the putative target genes were sent to Gene Ontology and Kyoto Encyclopedia of Genes and Genomes pathway analyses.

**Results:**

MiR-198-5p was low expressed in LUSC tissues. The combined standard mean difference (SMD) values of miR-198-5p expression based on GEO datasets were − 0.30 (95% confidence interval (CI) − 0.54, − 0.06) and − 0.39 (95% CI − 0.83, 0.05) using fixed effect model and random effect model, respectively. The sensitivity and specificity were not sufficiently high, as the area under the curve (AUC) was 0.7749 (Q* = 0.7143) based on summarized receiver operating characteristic (SROC) curves constructed using GEO datasets. Based on the in-house RT-qPCR, miR-198-5p expression was 4.3826 ± 1.7660 in LUSC tissues and 4.4522 ± 1.8263 in adjacent normal tissues (*P* = 0.885). The expression of miR-198-5p was significantly higher in patients with early TNM stages (I-II) than that in cases with advanced TNM stages (III-IV) (5.4400 ± 1.5277 vs 3.5690 ± 1.5228, *P* = 0.008). Continuous variable-based meta-analysis of GEO and PCR data displayed the SMD values of − 0.26 (95% CI − 0.48, − 0.04) and − 0.34 (95% CI − 0.71, 0.04) based on fixed and random effect models, respectively. As for the diagnostic value of miR-198-5p, the AUC based on the SROC curve using GEO and PCR data was 0.7351 (Q* = 0.6812). In total, 542 genes were identified as the targets of miR-198-5p. The most enriched Gene Ontology terms were epidermis development among biological processes, cell junction among cellular components, and protein dimerization activity among molecule functions. The pathway of non-small cell lung cancer was the most significant pathway identified using Kyoto Encyclopedia of Genes and Genomes analysis.

**Conclusion:**

The expression of miR-198-5p is related to the TNM stage. Thus, miR-198-5p might play an important role via its target genes in LUSC.

## Background

Lung cancer ranks first among all cancers in terms of incidence, and it is also the most important cause of cancer death all over the world [1]. Non-small cell lung cancer (NSCLC) accounts for around 85% of all lung cancers, with 30% of NSCLC cases being classified as lung squamous cell carcinoma (LUSC) [2–7]. Many patients are diagnosed with NSCLC at an advanced phase, which is attributable for the high mortality [8]. Therefore, more effective biomarkers are urgently needed in LUSC.

MicroRNAs (miRNAs) are ∼ 22-nt long endogenous RNAs that play significant roles in various cellular processes [9]. Previous studies have shown that miRNAs can target mRNAs involved in most of the developmental processes and are thus associating with many diseases [10]. Moreover, miRNAs have also been found to be involved in cancer [11]. Studies have found that miR-198-5p plays a vital role in many human cancers, including lung cancer [12]. A previous study [12] investigated the relationship between FGFR1 and miR-198-5p. However, the expression pattern of miR-198-5p in LUSC remains unknown. Additionally, the prospective target genes of miR-198-5p in LUSC have not yet been identified. Therefore, the relationship between miR-198-5p and lung squamous cell carcinoma as well as the underlying mechanism remains unknown.

To evaluate the clinical significance of miR-198-5p in LUSC, we examined miR-198-5p expression in LUSC tissues and carried out additional specific analyses to uncover its clinicopathological role. Furthermore, we performed big data analysis based on the Gene Expression Omnibus (GEO) and the Cancer Genome Atlas (TCGA). Subsequently, bioinformatics examinations were conducted to investigate the probable mechanism of miR-198-5p in LUSC.

## Methods

### Retrieval of data and publications from TCGA and GEO

**Fig. 1:**
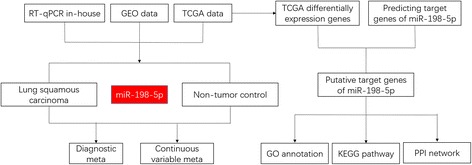
Flowchart representing the main design of our study

The flowchart representing the main design of our study is shown in Fig. 1. We downloaded miRNA expression data from the Cancer Genome Atlas (TCGA) associated with LUSC. All data were converted to a log2 scale. We also retrieved data from the Gene Expression Omnibus (GEO) and ArrayExpress to assess the expression pattern of miR-198-5p in LUSC and corresponding non-tumor samples. The search terms were as follows: (“lung” OR “pulmonary” OR “respiratory” OR “bronchioles” OR “bronchi” OR “alveoli” OR “pneumocytes” OR “air way” [MeSH]) AND (“cancer” OR “carcinoma” OR “tumor” OR “neoplas” OR “malignan” “squamous cell carcinoma” OR “adenocarcinoma” [MeSH]) OR/AND (“MicroRNA” OR “miRNA” OR “MicroRNA” OR “Small Temporal RNA” OR “noncoding RNA” OR “ncRNA” OR “small RNA” [MeSH]). Datasets with expression levels of miR-198-5p in LUSC and corresponding non-tumor samples were included. Other types of tumor or other miRNA were excluded. The number of samples in the tumor and non-tumor groups was at least three. The expression level of miR-198-5p in the datasets was converted to a log2 scale. The number, mean, and standard deviation of miR-198-5p levels in the tumor and non-tumor groups were calculated. We also searched PubMed, Web of Science, Science Direct, Google Scholar, Ovid, LILACS, Wiley Online Library, EMBASE, Cochrane Central Register of Controlled Trials, Chong Qing VIP, CNKI, Wan Fang, and China Biology Medicine disc; however, no publications regarding miR-198-5p expression in LUSC were found in these databases.

### RT-qPCR

After the formalin-fixed, paraffin-embedded (FFPE) sections were dewaxed, total RNA was achieved from these sections with the miRNeasy FFPE Kit (QIAGEN) according to manufacturer’s instruction. The concentration of RNA was measured using Nanodrop 2000. MiR-191 (CAACGGAAUCCCAAAAGCAGCU) and miR-103 (AGCAGCAUUGUACAGGGCUAUGA) were used as stably expressed control miRNAs as previously reported [13]. Applied Biosystems 7900 PCR system was used to perform real-time quantitative PCR and detect miR-198-5p expression (GGUCCAGAGGGGAGAUAGGUUC). The relative expression of miR-198-5p was calculated using the formula 2^−ΔCq^.

### Statistical analysis

Paired sample *t* test and independent sample *t* test were performed using SPSS 23.0 to determine the association between miR-198-5p expression and various clinicopathological parameters based on real-time RT-qPCR and microarray data. *P* < 0.05 was regarded as being statistically significant. Receiver operating characteristic (ROC) curves were constructed using SPSS 23.0.

Concerning the meta-analysis based on all accessible data, we used Stata 14 to determine the combined expression value of miR-198-5p in tumor and non-tumor groups and its relationship with both standard mean difference (SMD) and summarized receiver operating characteristic (SROC). The fixed effect model was initially used. The random effect model was used when heterogeneity was detected. The data were considered heterogeneous when *I*^2^ > 50%. Subgroup analysis and sensitivity analysis were carried out to find out the source of heterogeneity. We tested publication bias with funnel plots. SPSS 23.0 was employed to explore the diagnostic value of miR-198-5p in LUSC based on GEO data. Then, we performed a diagnostic meta-analysis using MetaDiSc 1.4. Meta-regression and threshold effect analysis were performed to determine the source of heterogeneity. The data from TCGA were not included in the analysis because of missing data.

### Differentially expressed mRNAs in LUSC based on TCGA

The expression level of each mRNA transformed into the log2 scale was evaluated using DESeq R package. We obtained 9860 differentially expressed genes in LUSC, including 6092 upregulated and 3768 downregulated genes.

### Selection of putative target genes of miR-198-5p

Predictions were conducted in silico with miRWalk 2.0 (http://zmf.umm.uni-heidelberg.de/apps/zmf/mirwalk2/). Genes that were present in more than 5 of the 12 prediction online tools were selected for further analysis. The selected genes were cross-referenced with the differentially expressed genes in TCGA. The overlapping genes were considered the putative targets of miR-198-5p in LUSC.

### Bioinformatics analyses

Gene Ontology (GO) annotation via DAVID (https://david.ncifcrf.gov/) was performed, including biological processes (BP), cellular components (CC), and molecular functions (MF). The Kyoto Encyclopedia of Genes and Genomes (KEGG) pathways associated with the putative target genes were also analyzed in DAVID. The results of GO annotation and KEGG pathway analysis were visualized using BiNGO and EnrichmentMap plugins in Cytoscape version 3.5.0. We used STRING (https://string-db.org/) to build interaction maps of the proteins encoded by the putative target genes.

### Validation of the putative target genes in the most significant KEGG pathway based on TCGA data

We selected the genes in the most significant KEGG pathway “non-small cell lung cancer” for further analysis. The differences in the expression levels of E2F2, E2F3, TGFA, PRKCG, CDK6, EGF, and CDK4 between LUSC and non-tumor tissues were analyzed based on TCGA data.

## Results

### GEO data mining to determine the expression and diagnostic value of miR-198-5p

**Fig. 2:**
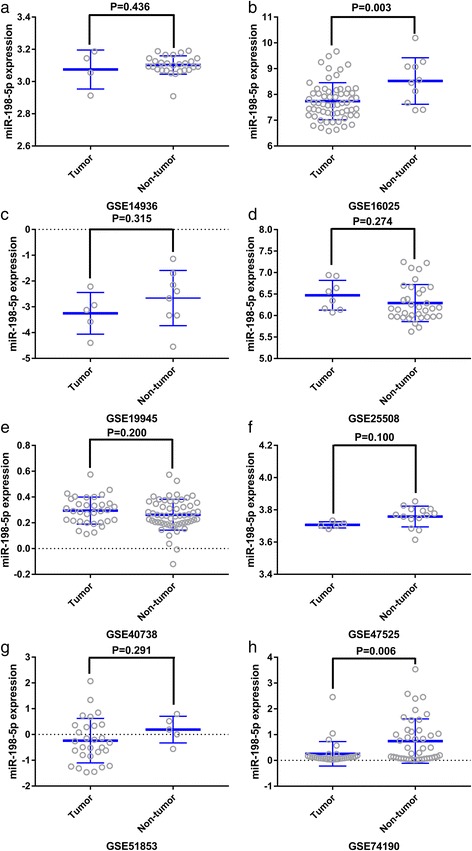
Scatterplots based on the included GEO datasets. **a** GSE14936. **b** GSE16025. **c** GSE19945. **d** GSE25508. **e** GSE40738. **f** GSE47525. **g** GSE51853. **h** GSE74190

**Fig. 3:**
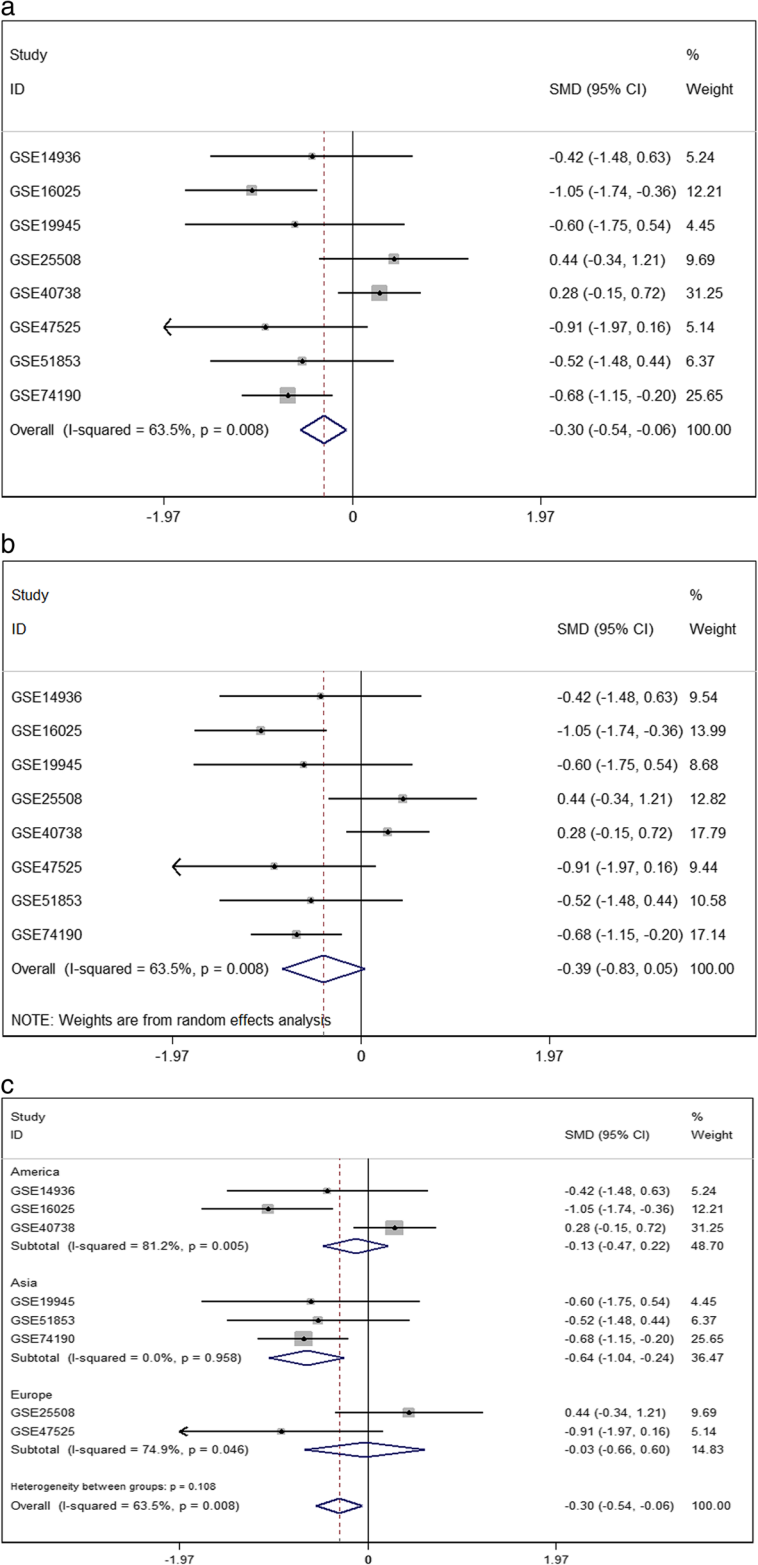
Continuous variable meta-analysis based on GEO datasets. **a** Forest plot based on fixed effect model **b** Forest plot based on random effect model **c** Subgroup analysis by region

**Fig. 4:**
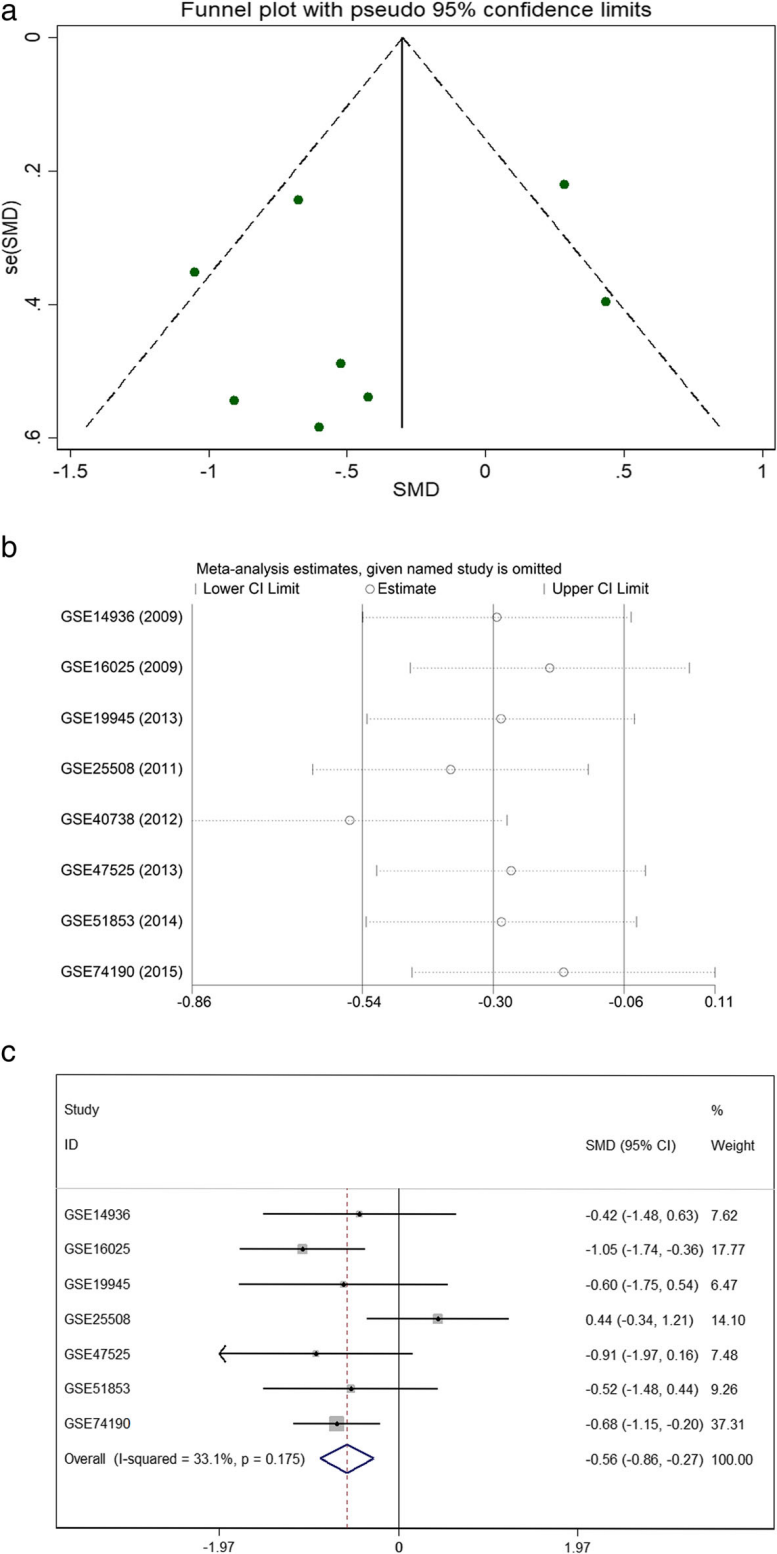
Continuous variable meta-analysis based on GEO datasets. **a** Funnel plot. **b** Sensitivity analysis. **c** Forest plot after the elimination of GSE40738

**Fig. 5:**
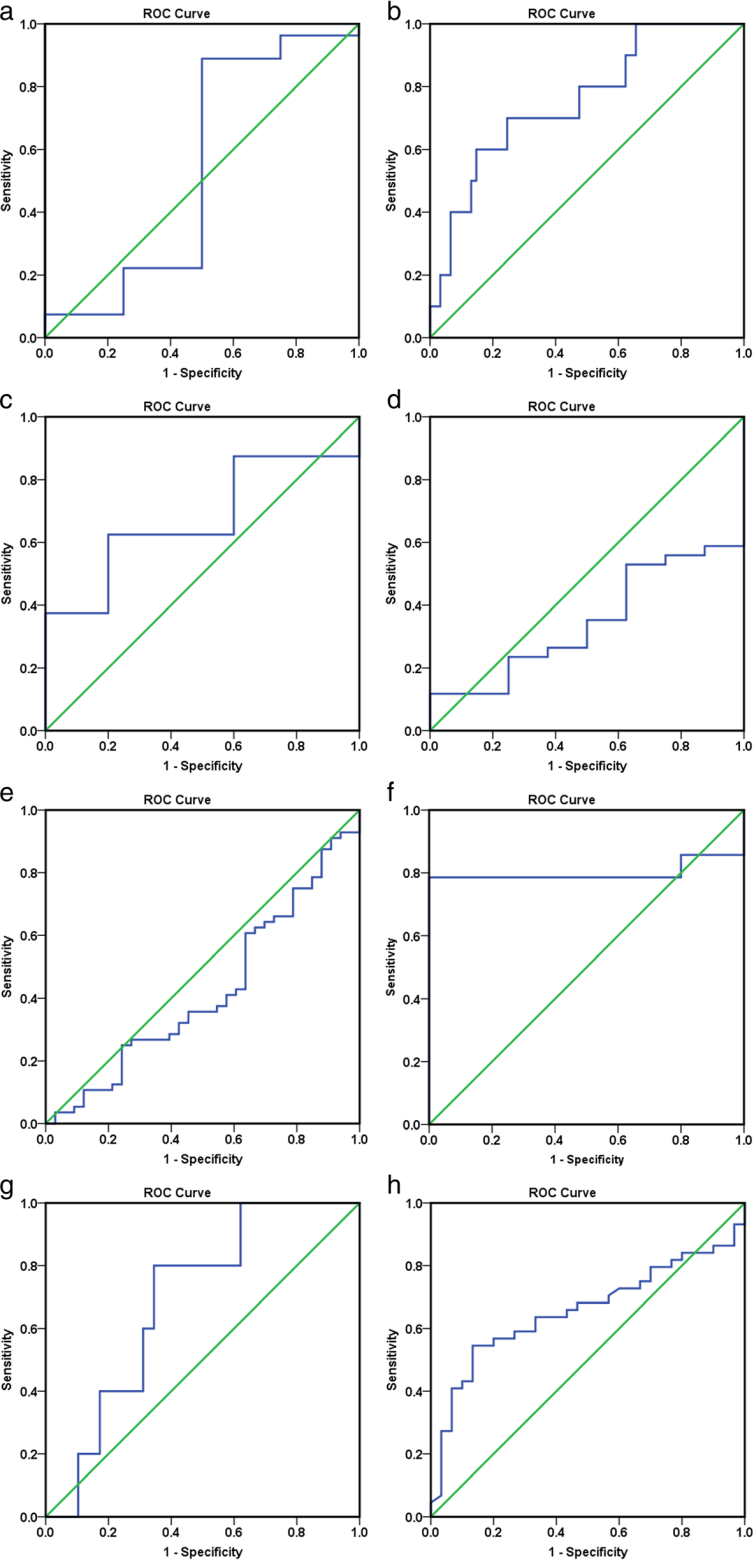
Receiver operating characteristic (ROC) curves based on GEO datasets. **a** GSE14936. **b** GSE16025. **c** GSE19945. **d** GSE25508. **e** GSE40738. **f** GSE47525. **g** GSE51853. **h** GSE74190

**Fig. 6:**
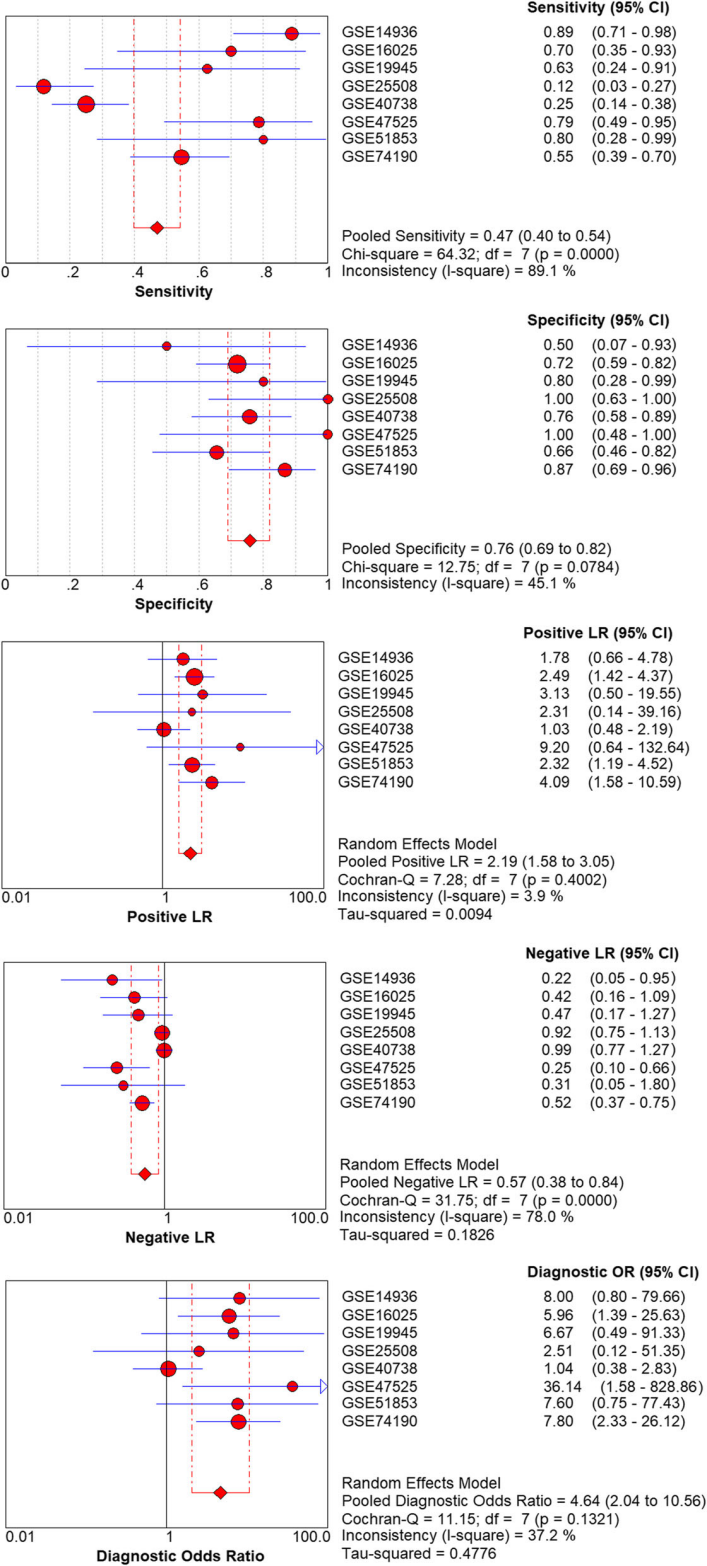
Pooled sensitivity, specificity, positive likelihood ratio, negative likelihood ratio, diagnostic score, and odds ratio obtained using MetaDisc 1.4 based on GEO datasets

**Fig. 7:**
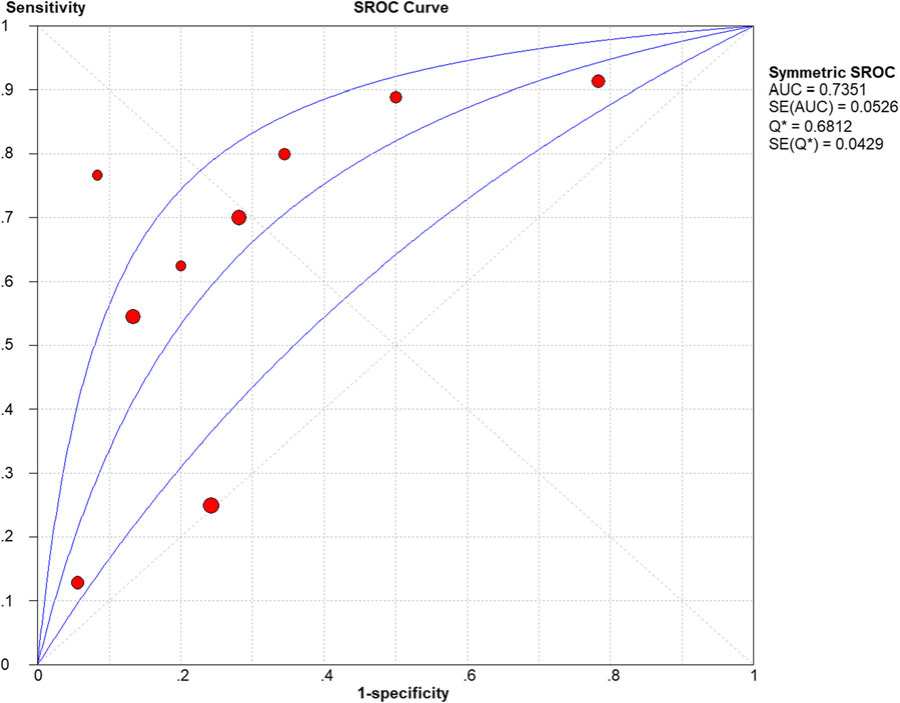
Summarized receiver operating characteristic (SROC) curve of GEO datasets

Considering the meta-analysis of miR-198-5p expression-based GEO data, eight datasets were included in our study. The scatter plots based on the GEO datasets are shown in Fig. 2. Forest plots using both fixed effect model (Fig. 3a) and random effect model (Fig. 3b) represented the expression level of miR-198-5p in LUSC. The combined effect sizes were − 0.30 (95% CI − 0.54, − 0.06) and − 0.39 (95% CI − 0.83, 0.05) based on the fixed and random effect models, respectively. Subgroup analysis showed that there was no heterogeneity among the studies from Asia (*I*^2^ = 0.0%) (Fig. 3c). The corresponding funnel plot is shown in Fig. 4a (*P* > 0.05). Sensitivity analysis (Fig. 4b) showed that the dataset GSE40738 might be a source of heterogeneity. The forest plot after the removal of GSE40738 is shown in Fig. 4c. The adjusted combined SMD value was − 0.56 (95% CI − 0.86, − 0.27) with *I*^2^ = 33.1%. The receiver operating characteristic curves based on the included datasets from GEO database are shown in Fig. 5. The pooled sensitivity, specificity, positive likelihood ratio, negative likelihood ratio, and diagnostic odds ratio were 0.47 (95% CI 0.40, 0.54), 0.76 (95% CI 0.69, 0.82), 2.19 (95% CI 1.58, 3.05), 0.57 (95% CI 0.38, 0.84), and 4.64 (95% CI 2.04, 10.56), respectively (Fig. 6). The area under the curve (AUC) based on the summarized receiver operating characteristic (SROC) curve was 0.7749 (Q* = 0.7143) (Fig. 7). We did not find a threshold effect of miR-198-5p in the study (*P* = 0.058). Only study region was determined to be a covariant in the meta-regression, and thus it was likely not a source of heterogeneity (*P* = 0.0550) (Table 1).

**Table 1:** Meta-regression based on GEO data

Var	Coeff.	Std. Err.	*P* value	RDOR	[95% CI]
Cte.	0.123	0.782	0.8816	–	–
S	0.5	0.2152	0.0679	–	–
Region	1.221	0.4899	0.055	3.39	(0.96;11.95)

### Clinical value of miR-198-5p in LUSC assessed using RT-qPCR

**Fig. 8:**
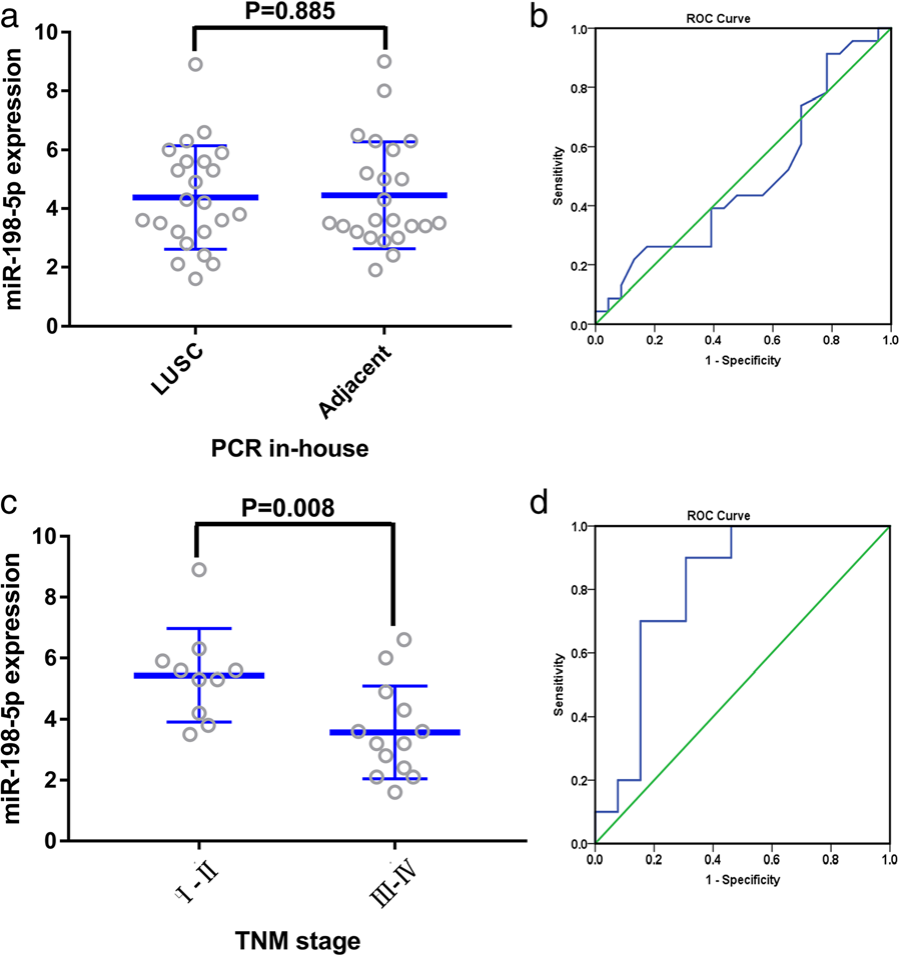
Relationship between miR-198-5p expression and TNM stage in lung squamous cell carcinoma (LUSC) and diagnostic value of miR-198-5p expression. Scatterplots (**a** LUSC and adjacent non-cancerous tissues and **c** TNM stage). Receiver operating characteristic (ROC) curves (**b** LUSC and adjacent non-cancerous tissues and **d** TNM stage)

Using RT-qPCR, the expression of miR-198-5p in the LUSC tissues was (4.3826 ± 1.7660) compared with that in the non-tumor tissues (4.4522 ± 1.8263, *P* = 0.885) (Fig. 8a, b). The other clinicopathological features of the LUSC case are shown in Table 2. Notably, the expression level of miR-198-5p in patients with early TNM stage (I-II) was (5.4400 ± 1.5277) compared to (3.5690 ± 1.5228) in patients with advanced TNM stage (III-IV) (*P* = 0.008) (Fig. 8c, d).

**Table 2:** Relationship between the expression of miR-198-5p and clinicopathological features in LUSC patients

Clinicopathological feature		*n*	miR-198-5p expression (2^−ΔCq^)		
			Mean ± SD	*t*	*P*
Tissue	LUSC	23	4.3826 ± 1.7660	0.146	0.885
	Non-tumor	23	4.4522 ± 1.8263		
Gender	Male	18	4.4670 ± 1.8711	0.425	0.675
	Female	5	4.0800 ± 1.4584		
Age	< 60	15	4.0930 ± 1.4270	− 1.080	0.293
	≥ 60	8	4.9250 ± 2.2833		
Smoking state	No	12	4.3000 ± 1.2884	− 0.229	0.821
	Yes	11	4.4730 ± 2.2401		
Size	≤ 3 cm	7	4.2570 ± 2.1196	− 0.220	0.828
	> 3 cm	16	4.4380 ± 1.6633		
EGFR amplification	No	17	4.7470 ± 1.8084	1.741	0.096
	Yes	6	3.3500 ± 1.2357		
Vascular invasion	No	20	4.6100 ± 1.6914	1.656	0.113
	Yes	3	2.8670 ± 1.7786		
TNM stage	I-II	10	5.4400 ± 1.5277	2.917	0.008
	III-IV	13	3.5690 ± 1.5228		
LNM	No	11	4.8730 ± 2.0553	1.294	0.210
	Yes	12	3.9330 ± 1.3918		
EGFR protein	Low	18	4.3280 ± 1.9013	− 0.277	0.785
	High	5	4.5800 ± 1.3142		
MET	Low	13	4.9230 ± 1.6559	1.750	0.095
	High	10	3.6800 ± 1.7313		
Grading	II	16	4.4130 ± 1.8326	0.12	0.906
	III	7	4.3140 ± 1.7411		

### Meta-analysis of miR-198-5p expression based on PCR and GEO data

**Fig. 9:**
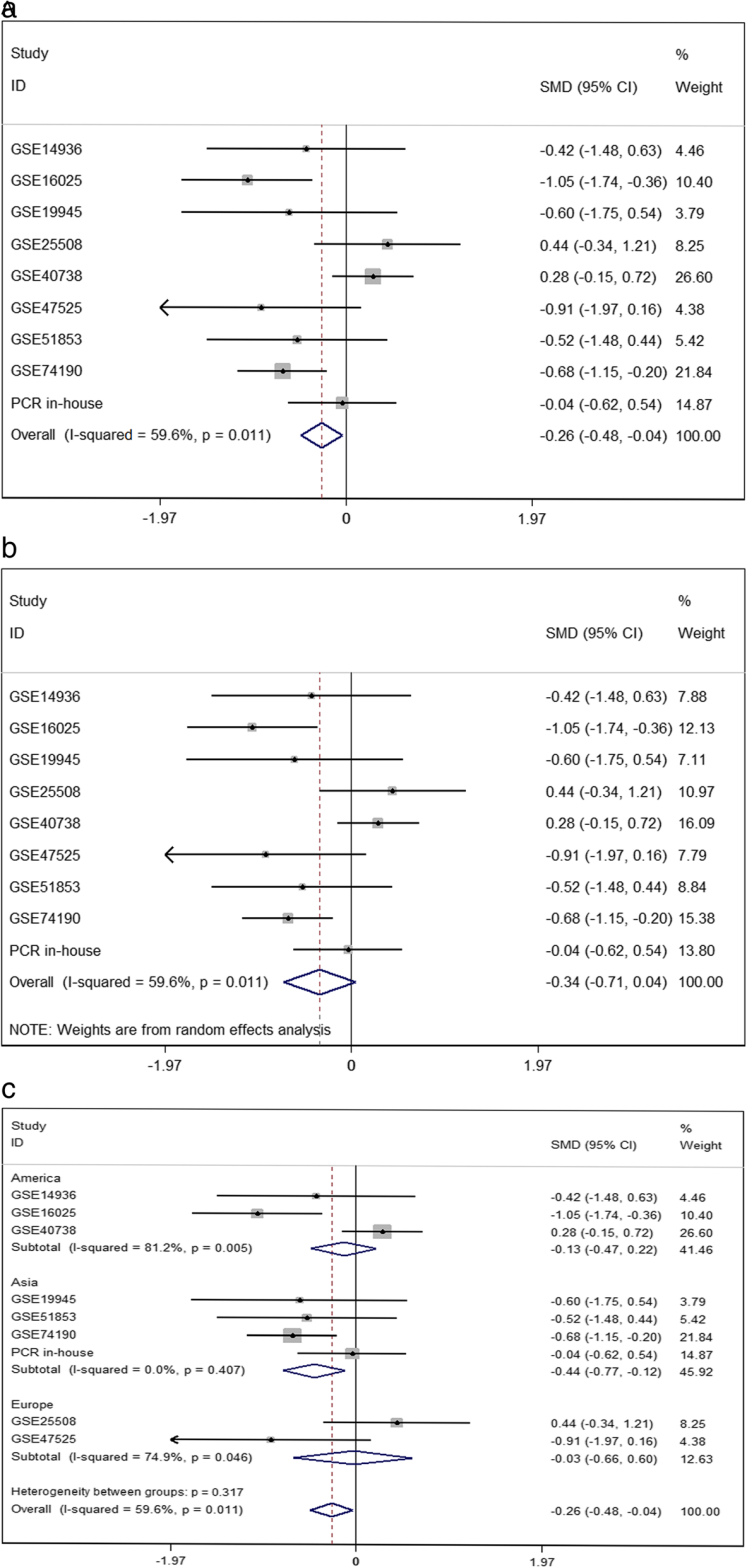
Continuous variable meta-analysis based on GEO datasets and in-house RT-qPCR results. **a**: Forest plot based on fixed effect model, (**b)**: forest plot based on random effect model, (**c)**: subgroup analysis by region

**Fig. 10:**
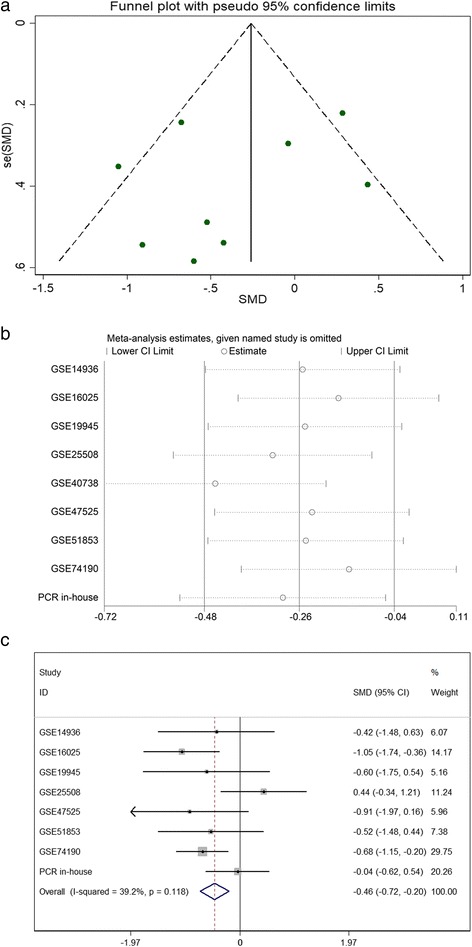
Continuous variable meta-analysis based on GEO datasets and in-house RT-qPCR results. **a** Funnel plot. **b** Sensitivity analysis. **c** Forest plot after the elimination of GSE40738

**Fig. 11:**
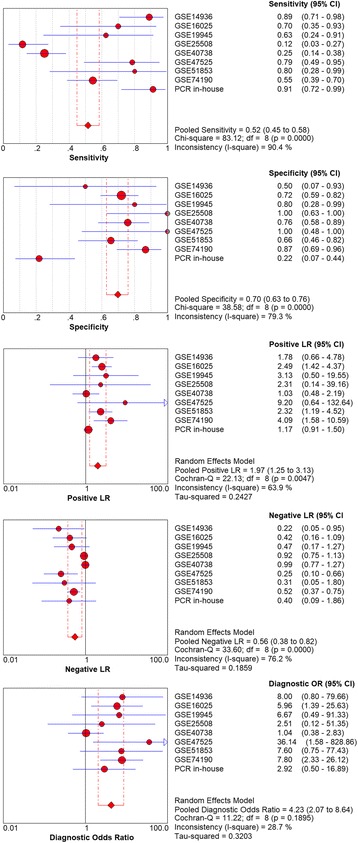
Pooled sensitivity, specificity, positive likelihood ratio, negative likelihood ratio, diagnostic score, and odds ratio obtained using MetaDisc 1.4 based on GEO datasets and in-house RT-qPCR results

**Fig. 12:**
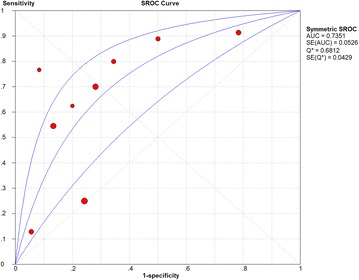
Summarized receiver operating characteristic (SROC) curve of GEO datasets and in-house RT-qPCR results

The combined SMD values based on GEO and PCR data were − 0.26 (95% CI − 0.48, − 0.04) (Fig. 9a) and − 0.34 (95% CI − 0.71, 0.04) (Fig. 9b) using the fixed and random effect models, respectively. In subgroup analysis, no heterogeneity among the studies from Asia was observed (*I*^2^ = 0.0%) (Fig. 9c). The corresponding funnel plot is shown in Fig. 10a (*P* > 0.05). Once again, study region and the dataset GSE40738 were identified as sources of heterogeneity (Fig. 10b). After eliminating GSE40738 from the analysis, the SMD changed to − 0.46 (95% CI − 0.72, − 0.20) (Fig. 10c). The pooled sensitivity, specificity, positive likelihood ratio, negative likelihood ratio, and diagnostic odds ratio were 0.52 (95% CI 0.45, 0.58), 0.70 (95% CI 0.63, 0.76), 1.97 (95% CI 1.25, 3.13), 0.56 (95% CI 0.38, 0.82), and 4.23 (95% CI 2.07, 8.64), respectively (Fig. 11). The area under the curve (AUC) based on the summarized receiver operating characteristic (SROC) curve was 0.7351 (Q* = 0.6812) (Fig. 12). We also observed threshold effect of miR-198-5p in this study (*P* = 0.013). In this case, study region was not accountable for heterogeneity (*P* = 0.2107) (Table 3).

**Table 3:** Meta-regression based on GEO and in-house RT-qPCR data

Var	Coeff.	Std. Err.	*P* value	RDOR	[95%CI]
Cte.	0.27	0.9327	0.7816	–	–
S	0.125	0.1632	0.4714	–	–
Region	0.751	0.5363	0.2107	2.12	(0.57;7.88)

### Gene Ontology (GO), Kyoto Encyclopedia of Genes and Genomes (KEGG) pathway annotation, and protein-protein interaction (PPI) network

**Fig. 13:**
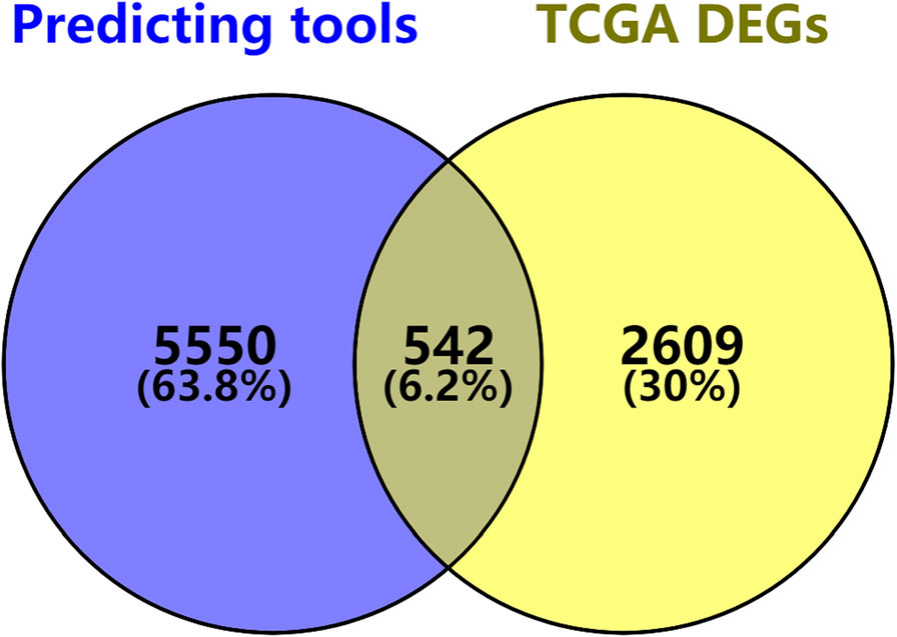
Venn diagram based on the 12 predicting tools and the differentially expressed genes (DEGs) in TCGA

**Fig. 14:**
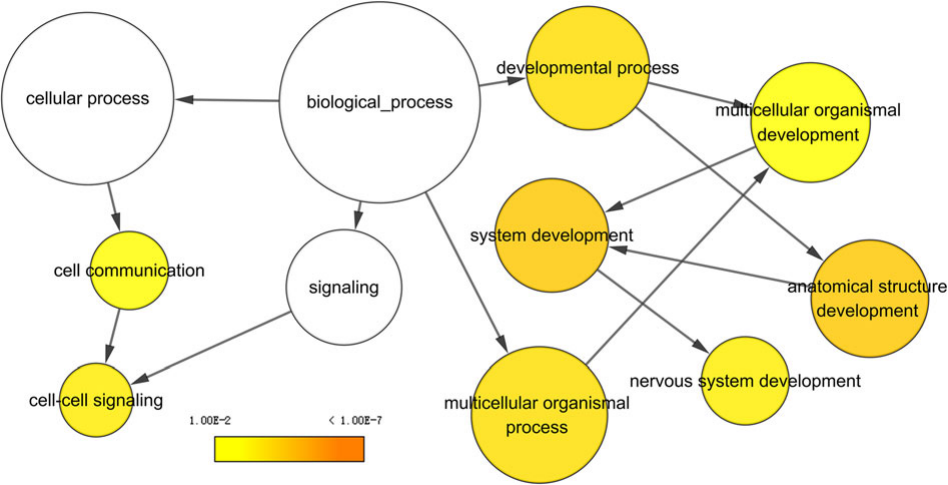
Biological processes (BP) were analyzed using the BiNGO plugin in Cytoscape

**Fig. 15:**
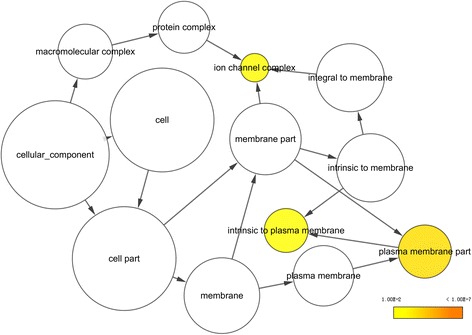
Cellular components (CC) were analyzed using the BiNGO plugin in Cytoscape

**Fig. 16:**
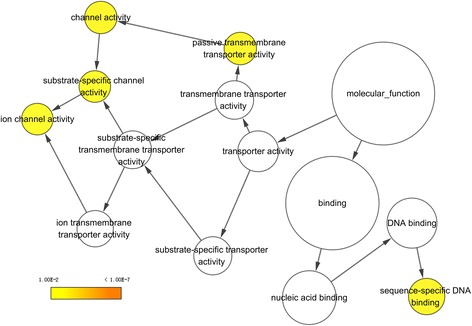
Molecular functions (MF) were analyzed using the BiNGO plugin in Cytoscape

**Fig. 17:**
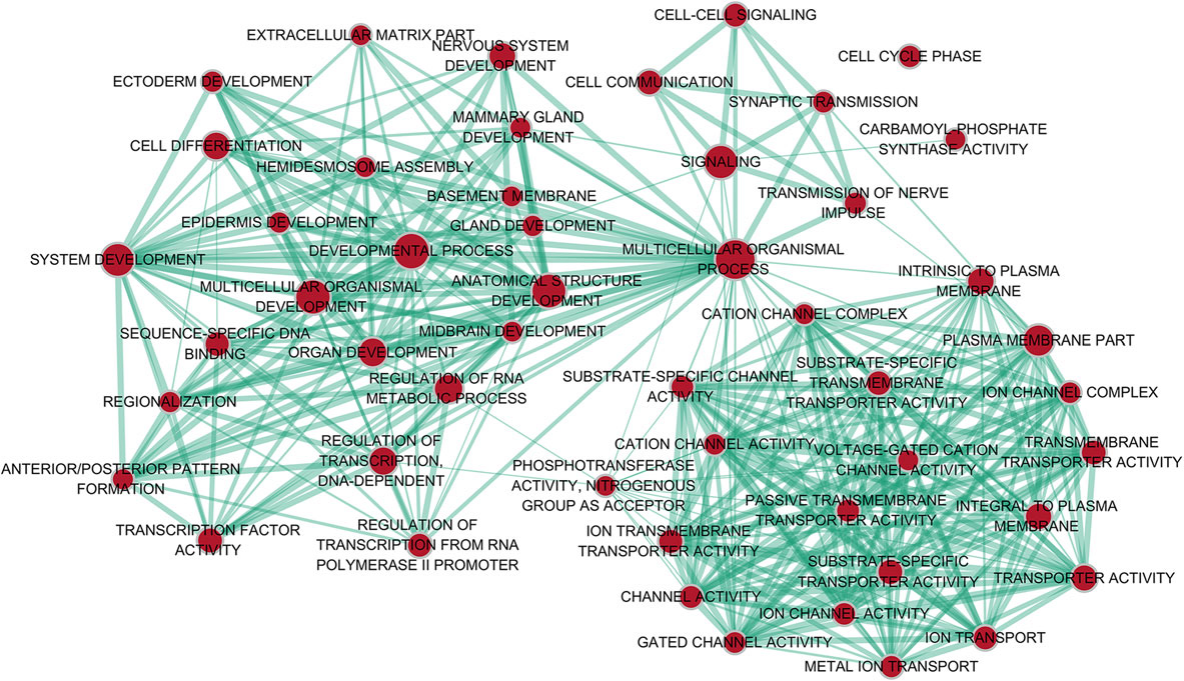
Kyoto Encyclopedia of Genes and Genomes (KEGG) pathway analysis was performed using the EnrichmentMap plugin in Cytoscape

**Fig. 18:**
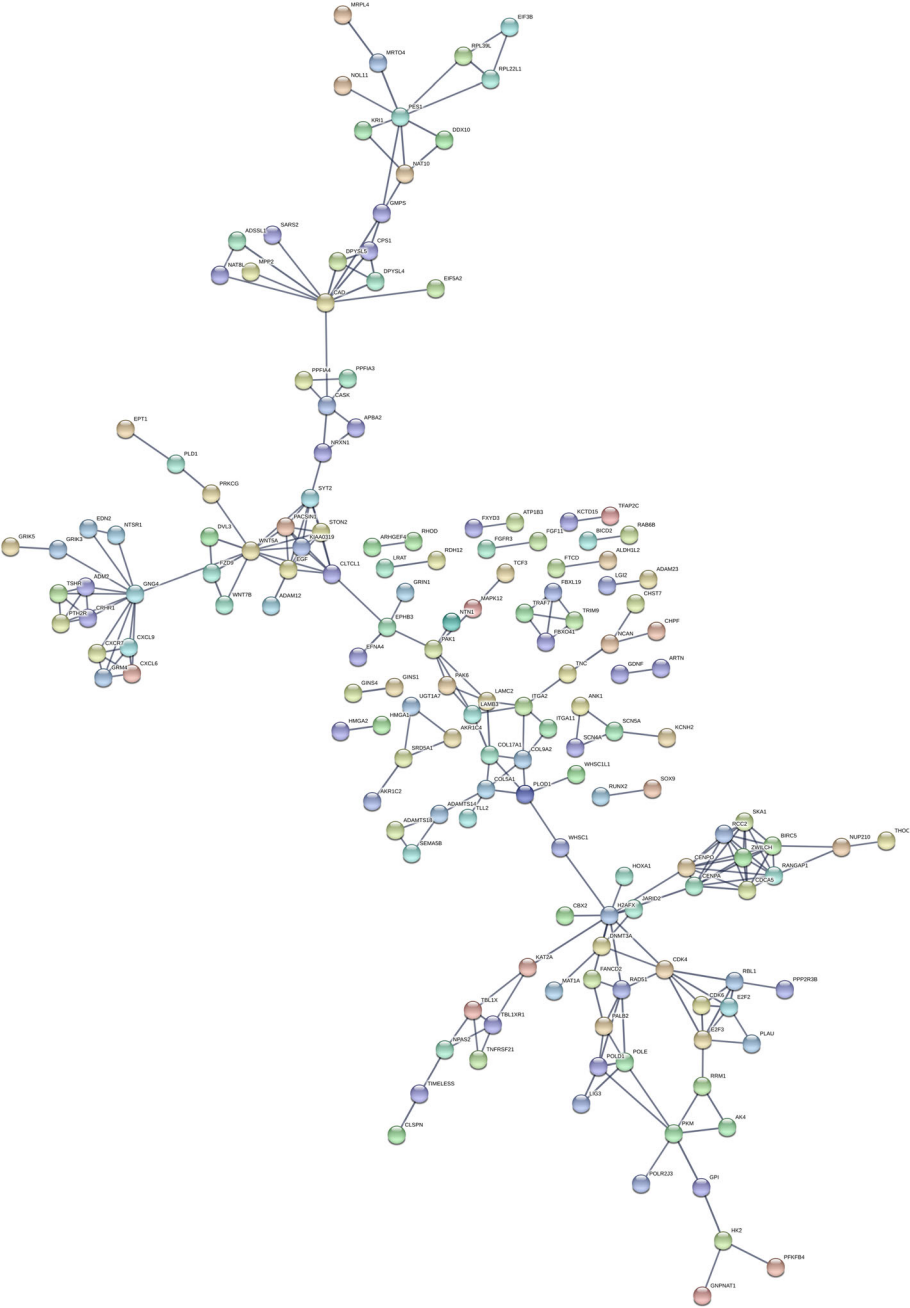
Protein-protein interaction (PPI) networks with 537 nodes and 848 edges were constructed using STRING. The PPI enrichment *P* value was 1.7E−14. Disconnected nodes were hidden in the network

**Fig. 19:**
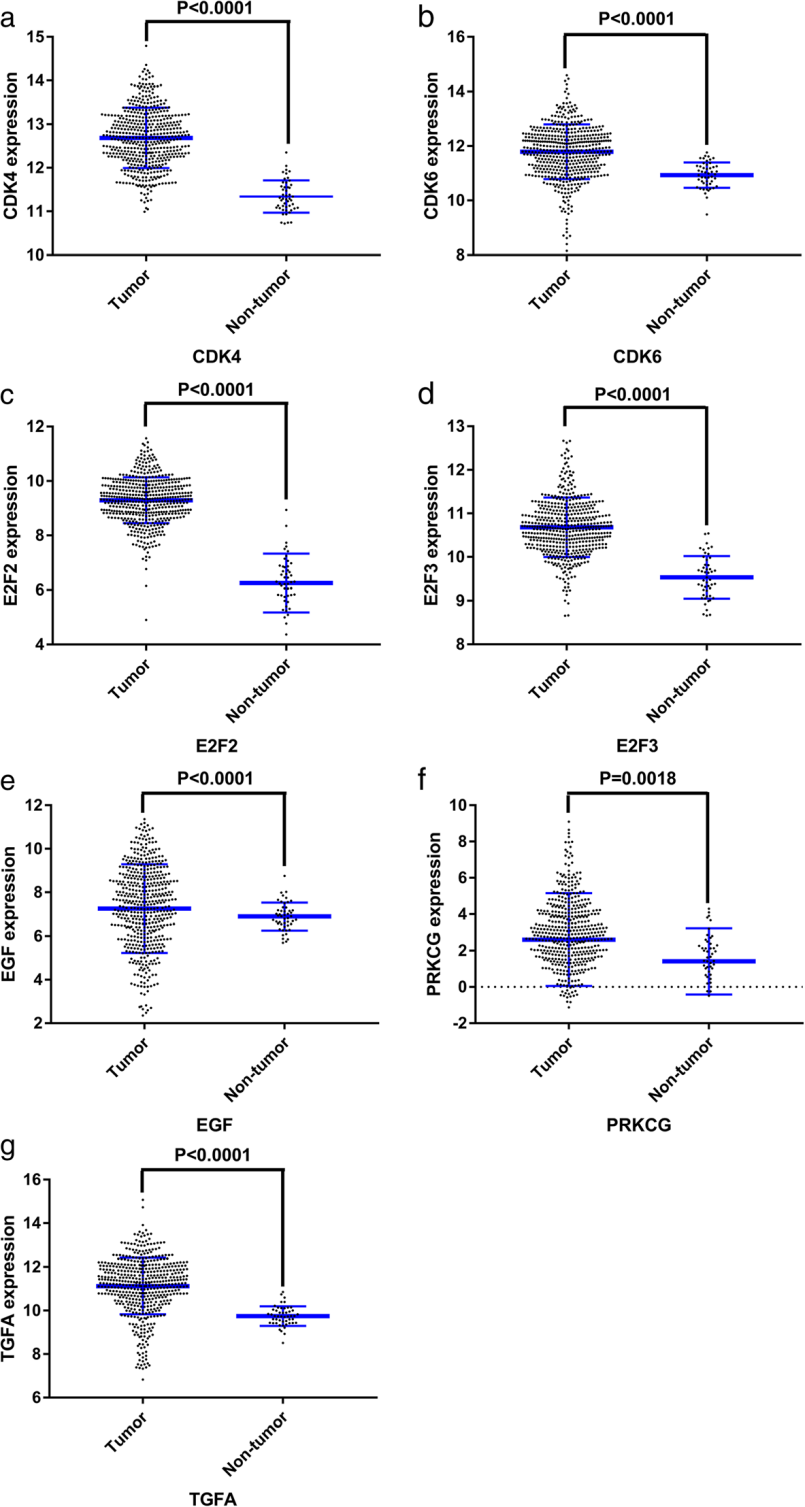
Scatterplots of the seven chosen genes from the Cancer Genome Atlas (TCGA). **a** CDK4. **b** CDK6. **c** E2F2. **d** E2F3. **e** EGF. **f** PRKCG. **g** TGFA

**Fig. 20:**
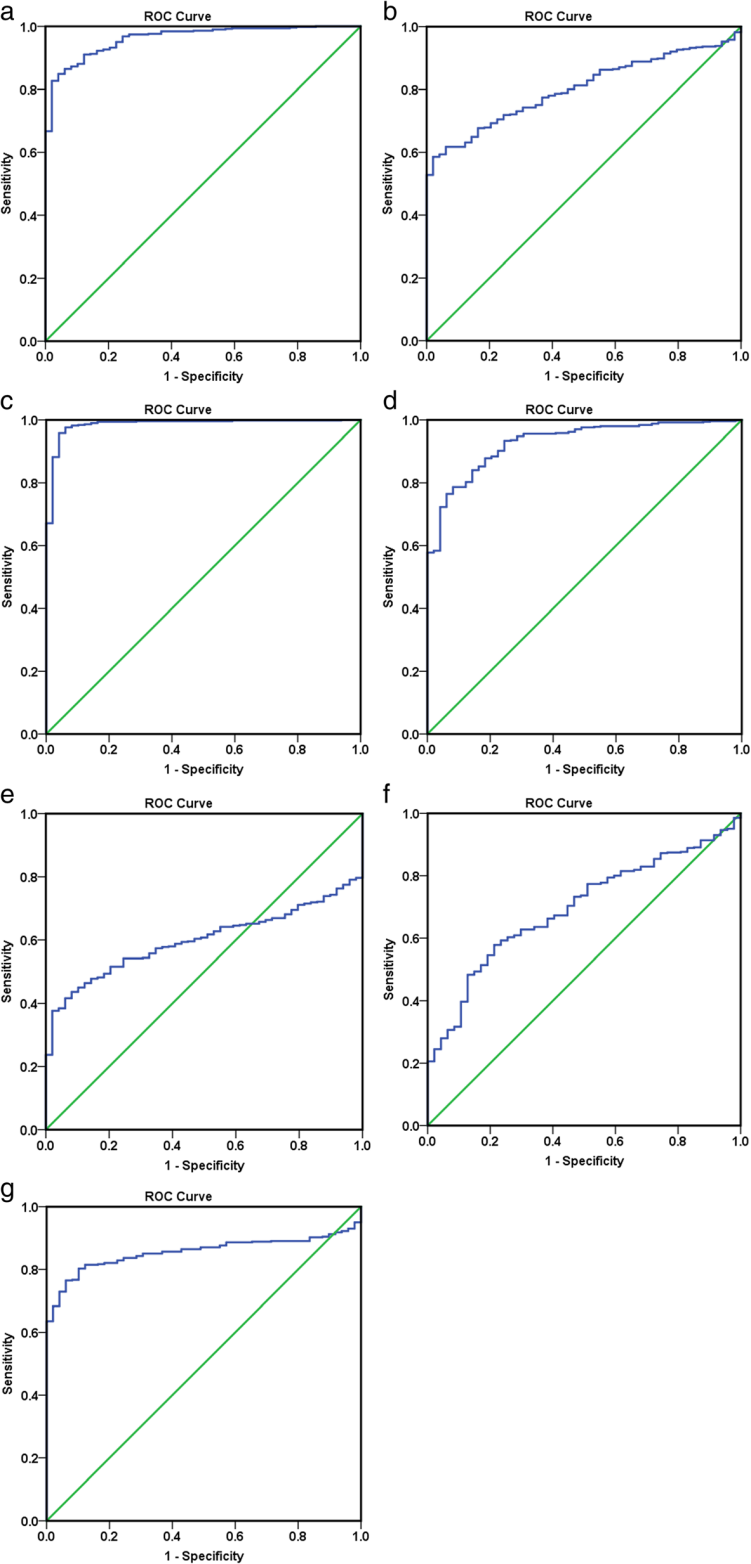
Receiver operating characteristic (ROC) curves of the seven chosen genes from the Cancer Genome Atlas (TCGA). **a** CDK4. **b** CDK6. **c** E2F2. **d** E2F3. **e** EGF. **f** PRKCG. **g** TGFA

In total, 542 genes were considered putative targets of miR-198-5p in LUSC (Fig. 13). Based on Gene Ontology (GO) analysis, the putative target genes of miR-198-5p were predominantly related to epidermis development with respect to biological processes (*P* = 4.84E−04) (Fig. 14, Table 4), cell junction in the case of cellular components (*P* = 7.60E−05) (Fig. 15, Table 4), and protein dimerization activity regarding molecular functions (*P* = 8.03E−05) (Fig. 16, Table 4). In terms of the KEGG pathway, the putative target genes of miR-198-5p were associated with non-small cell lung cancer, pathways in cancer and pancreatic cancer (Fig. 17, Table 5). The protein-protein interaction (PPI) network is shown in Fig. 18. The seven chosen genes in the KEGG pathway “non-small cell lung cancer” were all significantly upregulated in the LUSC samples compared to the non-tumor samples based on TCGA data (Fig. 19, Fig. 20).

**Table 4:** The most enriched 10 Gene Ontology (GO) term in biological process (BP) analysis, cellular component (CC), and molecular functions (MF)

Category	Term	Count	P value
GOTERM_BP_DIRECT	GO:0008544~epidermis development	10	4.84E−04
GOTERM_BP_DIRECT	GO:0043524~negative regulation of neuron apoptotic process	12	9.35E−04
GOTERM_BP_DIRECT	GO:0008284~positive regulation of cell proliferation	26	9.70E−04
GOTERM_BP_DIRECT	GO:0060070~canonical Wnt signaling pathway	9	0.001824
GOTERM_BP_DIRECT	GO:0007399~nervous system development	18	0.002211
GOTERM_BP_DIRECT	GO:0071277~cellular response to calcium ion	7	0.002457
GOTERM_BP_DIRECT	GO:0007269~neurotransmitter secretion	7	0.002457
GOTERM_BP_DIRECT	GO:0090103~cochlea morphogenesis	5	0.002641
GOTERM_BP_DIRECT	GO:0014075~response to amine	4	0.002773
GOTERM_BP_DIRECT	GO:0006366~transcription from RNA polymerase II promoter	26	0.003531
GOTERM_CC_DIRECT	GO:0030054~cell junction	29	7.60E−05
GOTERM_CC_DIRECT	GO:0005667~transcription factor complex	15	9.32E−04
GOTERM_CC_DIRECT	GO:0008021~synaptic vesicle	10	9.61E−04
GOTERM_CC_DIRECT	GO:0009986~cell surface	29	0.001109
GOTERM_CC_DIRECT	GO:0045202~synapse	13	0.004398
GOTERM_CC_DIRECT	GO:0005876~spindle microtubule	6	0.006912
GOTERM_CC_DIRECT	GO:0042734~presynaptic membrane	7	0.007013
GOTERM_CC_DIRECT	GO:0043195~terminal bouton	7	0.007013
GOTERM_CC_DIRECT	GO:0098793~presynapse	7	0.008171
GOTERM_CC_DIRECT	GO:0005887~integral component of plasma membrane	55	0.008929
GOTERM_MF_DIRECT	GO:0046983~protein dimerization activity	15	8.03E−05
GOTERM_MF_DIRECT	GO:0005509~calcium ion binding	36	9.88E−04
GOTERM_MF_DIRECT	GO:0016810~hydrolase activity, acting on carbon-nitrogen (but not peptide) bonds	4	0.00922
GOTERM_MF_DIRECT	GO:0005109~frizzled binding	5	0.017408
GOTERM_MF_DIRECT	GO:0001105~RNA polymerase II transcription coactivator activity	5	0.019099
GOTERM_MF_DIRECT	GO:0043565~sequence-specific DNA binding	24	0.019304
GOTERM_MF_DIRECT	GO:0008080~N-acetyltransferase activity	4	0.022413
GOTERM_MF_DIRECT	GO:0003682~chromatin binding	19	0.026956
GOTERM_MF_DIRECT	GO:0001077~transcriptional activator activity, RNA polymerase II core promoter proximal region sequence-specific binding	13	0.033582
GOTERM_MF_DIRECT	GO:0005262~calcium channel activity	6	0.045673

**Table 5:** Kyoto Encyclopedia of Genes and Genomes (KEGG) pathway annotation of the putative target genes of miR-198-5p

Category	Term	Count	*P* value
KEGG_PATHWAY	hsa05223:Non-small cell lung cancer	7	0.002996
KEGG_PATHWAY	hsa05200:Pathways in cancer	20	0.005776
KEGG_PATHWAY	hsa05212:Pancreatic cancer	7	0.006302
KEGG_PATHWAY	hsa05214:Glioma	7	0.006302
KEGG_PATHWAY	hsa04512:ECM-receptor interaction	8	0.00691
KEGG_PATHWAY	hsa04151:PI3K-Akt signaling pathway	17	0.015931
KEGG_PATHWAY	hsa04360:Axon guidance	9	0.016478
KEGG_PATHWAY	hsa05219:Bladder cancer	5	0.020529
KEGG_PATHWAY	hsa05222:Small cell lung cancer	7	0.021937
KEGG_PATHWAY	hsa04550:Signaling pathways regulating pluripotency of stem cells	9	0.027765
KEGG_PATHWAY	hsa05166:HTLV-I infection	13	0.032302
KEGG_PATHWAY	hsa05218:Melanoma	6	0.035606
KEGG_PATHWAY	hsa00230:Purine metabolism	10	0.037636
KEGG_PATHWAY	hsa04390:Hippo signaling pathway	9	0.040805
KEGG_PATHWAY	hsa03410:Base excision repair	4	0.05213
KEGG_PATHWAY	hsa00250:Alanine, aspartate and glutamate metabolism	4	0.060271
KEGG_PATHWAY	hsa05206:MicroRNAs in cancer	13	0.063619
KEGG_PATHWAY	hsa04014:Ras signaling pathway	11	0.066327
KEGG_PATHWAY	hsa04724:Glutamatergic synapse	7	0.073405
KEGG_PATHWAY	hsa05230:Central carbon metabolism in cancer	5	0.082196
KEGG_PATHWAY	hsa05033:Nicotine addiction	4	0.083009
KEGG_PATHWAY	hsa04510:Focal adhesion	10	0.084102

## Discussion

MiR-198-5p was clearly under-expressed in LUSC tissues in comparison with non-cancer lung tissues. The cases with LUSC in Asia expressed lower levels of miR-198-5p than did the healthy controls; however, the expression pattern in other regions was unclear. Our RT-qPCR indicated that the expression of miR-198-5p might be related to the tumor TNM stage, which suggests that miR-198-5p likely plays a role in tumor growth, lymph node metastasis, or distant metastasis. However, the downregulation of miR-198-5p was not obvious in our in-house RT-qPCR analysis. The diagnostic validation and meta-analysis based on GEO and RT-qPCR data indicated that miR-198-5p might be a biomarker of LUSC, but the sensitivity and specificity were not sufficiently high. Similarly, study region may be a source of heterogeneity, which suggested that the diagnostic screening might be suitable for Asian populations but not for others. Apart from LUSC, several studies have explored the expression pattern and mechanism of miR-198-5p in other diseases. MiR-198-5p has been reported to be upregulated in multiple myeloma [14], chronic pancreatitis or pancreatic ductal adenocarcinoma [15], Parkinson’s disease [16], esophageal cancer [17], preeclampsia [18], pancreatic adenocarcinoma, ampullary adenocarcinoma [19], lupus nephritis [20], retinoblastoma [21], anencephaly [22], and squamous cell carcinoma of tongue [23]. On the other hand, low expression of miR-198-5p has been found in prostate cancer [24], breast cancer [25], glioblastoma [26, 27], hepatocellular carcinoma [28], especially hepatitis C virus-associated hepatocellular carcinoma [29, 30], osteosarcoma [31], gastric cancer [32], colorectal cancer [33], pancreatic cancer [34], and respiratory syncytial virus (RSV) infection [35]. The overexpression of miR-198-5p has also been documented in CD8+ T cells in renal cell carcinoma [36]. In prostate cancer, a recent study indicated that miR-198-5p is targeted by the long noncoding RNA SChLAP1, leading to the activation of the MAPK1 pathway, thereby promoting cancer cell proliferation and metastasis [24]. Another study suggested that miR-198-5p may be involved in prostate cancer [37]. In hepatocellular carcinoma, miR-198-5p has been shown to target the HGF/c-MET pathway [38]. Several studies have revealed that the expression of miR-198-5p is greatly related to lymph node metastasis or distant metastasis in different malignant diseases, such as breast cancer [25], osteosarcoma [31], gastric cancer [32], and colorectal cancer [33]. Some studies have also shown that miR-198-5p is closely related to cell proliferation, apoptosis, and migration [12, 14, 39, 40]. The relationship between miR-198-5p and cancer prognosis is controversial [15, 17, 27, 32–34]. Thus, we investigated whether miR-198-5p plays an important role in biological processes in various diseases, both malignant and benign.

In the case of lung adenocarcinoma, two reports have verified that miR-198-5p is under-expressed [12, 41]. However, studies on the characteristic of miR-198-5p in LUSC are lacking. One study assessed the diagnostic significance of miR-198-5p in lung adenocarcinoma, with sensitivity = 71.1%, specificity = 95.2%, and AUC = 0.887 (95% CI 0.801, 0.945) [41]. Our study highlighted the diagnostic value of miR-198-5p in LUSC. Yang et al. showed that miR-198-5p was capable to suppress proliferation and promote apoptosis in lung cancer cells by targeting FGFR1 [12], and Wu et al. showed that miR-198-5p promotes apoptosis, represses cell proliferation, and leads to cell cycle arrest in lung adenocarcinoma cells by directly targeting SHMT1 [39]. Our study showed that expression pattern and diagnostic value of miR-198-5p varied according to the race of the patient population, which should be further validated in larger samples.

Although many studies use prediction tools to determine miRNA target genes, the inadequate number of available prediction tools can lead to unreliable data. We used 12 online prediction tools based on miRWalk 2.0, and this method had not been previously utilized in LUSC. The predicted genes were cross-referenced with the differentially expressed genes in TCGA, which further enhanced the specificity and accuracy of our investigation. Because miR-198-5p is downregulated in LUSC, we chose the upregulated genes from TCGA. Via bioinformatics analyses, the putative target genes of miR-198-5p were most significantly enriched in the KEGG pathways of non-small cell lung cancer, pathways in cancer, pancreatic cancer, glioma, and ECM-receptor interactions. For the GO biological processes, the putative target genes of miR-198-5p were involved in epidermis development, negative regulation of neuron apoptotic processes, positive regulation of cell proliferation, the canonical Wnt signaling pathway, and nervous system development, which indicated that the putative target genes might regulated epidermis proliferation, cell apoptosis, or carcinoma of nervous tissues. In addition, for the GO cellular components, the putative target genes of miR-198-5p were enriched in cell junction, transcription factor complex, synaptic vesicle, cell surface proteins, and synapses, which are related to migration, metastasis, and intercellular exchange of molecules. In terms of the GO molecular functions, the putative target genes of miR-198-5p were associated with protein dimerization activity, calcium ion binding, hydrolase activity on carbon-nitrogen (but not peptide) bonds, frizzled binding, and RNA polymerase II transcription co-activator activity. To verify the accuracy of our analysis, we selected several genes and determined its expression based on TCGA. The genes included the pathway non-small cell lung cancer were E2F2, E2F3, TGFA, PRKCG, CDK6, EGF, and CDK4, which were all expressed at significantly higher levels in LUSC tissues in comparison to that in the non-cancer group. Thus, these genes are probably the targets of miR-198-5p. The putative target genes of miR-198 should be validated further in the future.

## Conclusions

MiR-198-5p might be downregulated in LUSC, especially in Asia region. The putative target genes of miR-198-5p were closely related to tumorigenesis and progress in LUSC.

## Abbreviations

LUSC: Lung squamous cell carcinoma
GEO: Gene Expression Omnibus
FFPE: Formalin-fixed, paraffin-embedded
mRNA: Messenger RNA
miRNA: Micro RNA
TCGA: The Cancer Genome Atlas
AUC: Area under the curve
SROC: Summarized receiver operating characteristic
NSCLC: Non-small cell lung cancer
GO: Gene Ontology
KEGG: Kyoto Encyclopedia of Genes and Genomes
BP: Biological processes
CC: Cellular components
MF: Molecular functions
PPI: Protein-protein interaction
